# Verticillium wilt resistant and susceptible olive cultivars express a very different basal set of genes in roots

**DOI:** 10.1186/s12864-021-07545-x

**Published:** 2021-04-01

**Authors:** Jorge A. Ramírez-Tejero, Jaime Jiménez-Ruiz, Alicia Serrano, Angjelina Belaj, Lorenzo León, Raúl de la Rosa, Jesús Mercado-Blanco, Francisco Luque

**Affiliations:** 1 Department of Experimental Biology, Center for Advanced Studies in Olive Grove and Olive Oils, University of Jaén, 23071 Jaén, Spain; 2 Institute of Agricultural and Fishery Research and Training (IFAPA), Alameda del Obispo’ Center, Avda. Menéndez Pidal s/n, 14004 Córdoba, Spain; 3 Department of Crop Protection, Institute for Sustainable Agriculture (CSIC), Córdoba, Spain

**Keywords:** RNA-Seq, *Olea europaea* L., Roots, Transcriptome, *Verticillium dahliae*

## Abstract

**Background:**

Olive orchards are threatened by a wide range of pathogens. Of these, *Verticillium dahliae* has been in the spotlight for its high incidence, the difficulty to control it and the few cultivars that has increased tolerance to the pathogen. Disease resistance not only depends on detection of pathogen invasion and induction of responses by the plant, but also on barriers to avoid the invasion and active resistance mechanisms constitutively expressed in the absence of the pathogen. In a previous work we found that two healthy non-infected plants from cultivars that differ in *V. dahliae* resistance such as ‘Frantoio’ (resistant) and ‘Picual’ (susceptible) had a different root morphology and gene expression pattern. In this work, we have addressed the issue of basal differences in the roots between Resistant and Susceptible cultivars.

**Results:**

The gene expression pattern of roots from 29 olive cultivars with different degree of resistance/susceptibility to *V. dahliae* was analyzed by RNA-Seq. However, only the Highly Resistant and Extremely Susceptible cultivars showed significant differences in gene expression among various groups of cultivars. A set of 421 genes showing an inverse differential expression level between the Highly Resistant to Extremely Susceptible cultivars was found and analyzed. The main differences involved higher expression of a series of transcription factors and genes involved in processes of molecules importation to nucleus, plant defense genes and lower expression of root growth and development genes in Highly Resistant cultivars, while a reverse pattern in Moderately Susceptible and more pronounced in Extremely Susceptible cultivars were observed.

**Conclusion:**

According to the different gene expression patterns, it seems that the roots of the Extremely Susceptible cultivars focus more on growth and development, while some other functions, such as defense against pathogens, have a higher expression level in roots of Highly Resistant cultivars. Therefore, it seems that there are constitutive differences in the roots between Resistant and Susceptible cultivars, and that susceptible roots seem to provide a more suitable environment for the pathogen than the resistant ones.

**Supplementary Information:**

The online version contains supplementary material available at 10.1186/s12864-021-07545-x.

## Background

Cultivated olive tree (*Olea europaea L. subsp*. *europaea* var. *europaea*) is one of the top worldwide-extended fruit tree crops, with a decisive economic impact especially in Mediterranean countries. Although it can be used as a source of different materials [1, 2], the main product of this fruit tree is extra virgin olive oil. This oil has been proven as highly beneficial food for human health in many studies [3] and its production also has a direct effect on circular economy via by-product exploitation, or even tourism promotion [4, 5]. Thus, all efforts that address olive tree cultivation improvement and protection must be considered essential in agriculture sustainability.

At present, several pathogens endanger olive tree cultivars all over the world. Of these, the pathogenic soil-borne fungus *Verticillium dahliae* Kleb. has been in the spotlight for the last two decades [6–8]. The disease caused by this pathogen (Verticillium wilt) and has dramatic consequences for trees and, depending on the infecting pathotype virulence, could end in complete defoliation and plant death [9]. The successful control of this disease needs integrated management strategy, including the use of cultivars with high resistance levels [9]. Unfortunately, most of the cultivars used today are susceptible to Verticillium wilt, and only a few resistant ones have been found, such as ‘Frantoio’ [9]. Therefore, finding new olive cultivars that are tolerant to this disease is extremely necessary [10]. For this purpose, knowing the genetic control of resistance to Verticillium wilt may be extremely important for speeding up the breeding selection process. In fact, previous works have shown that a systemic response of the resistant ‘Frantoio’ cultivar to *V. dahliae* inoculation in aerial tissues reveals an association between gene expression patterns of *GRAS1* and *DRR2* and resistance to this pathogen [8]. Furthermore, the differential gene expression between ‘Frantoio’ and ‘Picual’ has been observed not only in response to *V. dahliae* infection, but also in roots of uninfected healthy plants [11]. Infection by *V. dahliae* in ‘Picual’ roots causes a marked genetic response in early stages, and promotes the expression of those genes involved in plant defense and protein turnover [12]. These results suggest that differences in the expression profile, especially of roots, may be relevant for each cultivar’s susceptibility to this infection. It has also been determined that, regardless of the external symptom expression observed in olive cultivars, the pathogen is able to penetrate their roots and spread through plant tissues [10, 13]. However, olive cultivars show a differentiated ability to avoid the development of disease symptoms, which could correspond to the degree of differential resistance/susceptibility. Considering these previous findings, variability in the resistance level might be defined, at least partially, by differences in the gene expression pattern in roots not only in response to the pathogen but also prior to infection. In order to address the issue that basal differences in roots may be relevant for the infection process and susceptibility of the plant, this work included a transcriptomic study to determine the differential gene expression in roots of healthy plants of a wide variety of cultivars with different susceptibilities to *V. dahliae* infection.

## Results

### Differential gene expression among groups

The peer comparison between the disease resistance groups showed a large number of differentially expressed genes in roots between cultivars Highly Resistant (HR) and Extremely Susceptible (ES) compared to the intermediate groups of disease response (Table 1). By setting the threshold at 1% of False Discovery Rate (FDR) and any Fold Change (FC), the expression pattern in the roots of cultivars HR differed in 255 unique genes with resistant (R) group, 3883 with Moderately Susceptible (MS) group, 1161 with Susceptible (S) group and 418 with group ES (Table 1). The comparison of the expressed genes in roots of the ES cultivars displayed a similar trend, with 507 differentially expressed genes compared to group S, 3100 compared to group MS and 223 to group R. However, very few genes were differentially expressed at any FC among groups S, MS and R. In fact, a single gene was differentiated between groups R and MS, five genes between groups R and S and nine genes between groups MS and S (Table 1). Consequently, a more in-depth analysis was carried out for the gene expression in the roots of cultivars HR and ES.

**Table 1:** Matrix of the differentially expressed genes among groups. Statistical significance set at the 0.01 adjusted *p* value and False Discovery Rate (FDR) < 1%

	HR	R	MS	S	ES
**HR**	–	255	3883	1161	418
**R**		–	1	5	223
**MS**			–	9	3100
**S**				–	507
**ES**					–

*HR* highly-resistant, *R* resistant, *MS* moderately-susceptible, *S* susceptible, *ES* extremely-susceptible

### Differential gene expression profile of the HR and ES olive cultivars

**Fig. 1:**
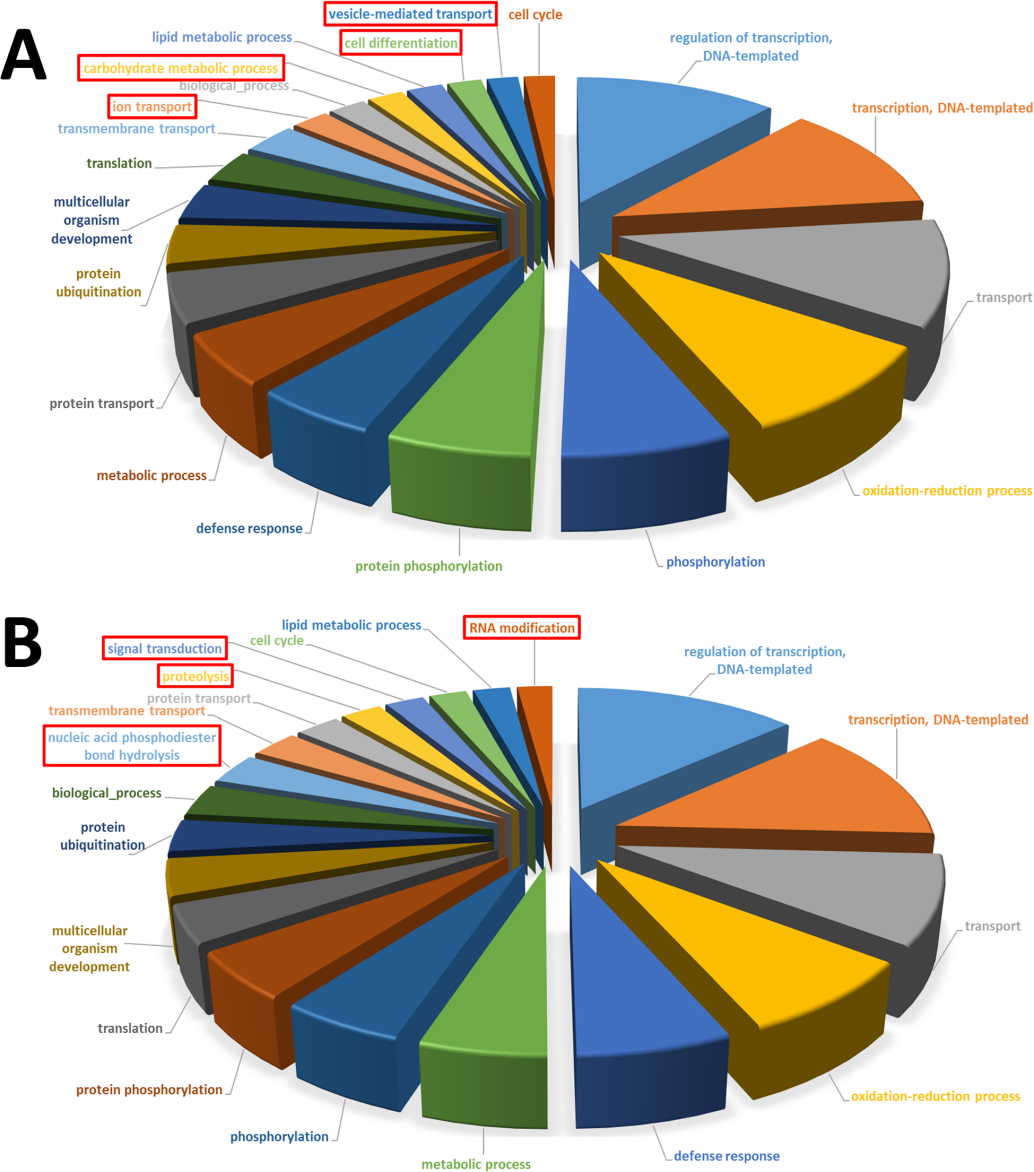
Top 20 Biological Processes at level 7 related with genes up regulated (**a**) and down regulated (**b**) in cultivars HR. Red boxes highlight terms that differ between both groups

The expression profile in the roots of cultivars HR was compared to that of the remaining groups (R-MS-S-ES) to, thus, identify 2942 unique genes differentially expressed with an 8 FC, or higher, and an FDR lower than 1%. These differentially expressed genes were classified in 1542 up-regulated and 1400 down-regulated genes in the roots of cultivars HR (see Additional file 1). They were analyzed by two different approaches. First, to obtain a global picture of the processes related to this new set, a Gene Ontology (GO) direct count of Biological Process (BP) was carried out on both the up- and down-regulated genes. As a result, a very similar list of terms was obtained given the presence of some critical processes, such as DNA transcription, transport or oxidation-reduction reactions (Fig. 1). Second, to evaluate the biological relevance of these gene sets compared to the whole root transcriptome, a GO terms enrichment analysis was conducted of both groups separately. By this approach, the up-regulated genes in the roots of cultivars HR were associated with the terms related to the nucleus and molecule transport was related to this organelle, such as nuclear envelop (GO:0005635), nuclear pore (GO:0005643), nucleocytoplasmic transport (GO:0006913), nuclear transport (GO:0051169) or protein localization to the nucleus (GO:0034504; Fig. 2). Only one of the enriched terms (ent-copalyl diphosphate synthase activity: GO:0009905) was not related to protein mobilization. Strikingly, the down-regulated genes did not show any enriched terms even though both groups were similar in size, which highlights the low specificity level of this gene set.

**Fig. 2:**
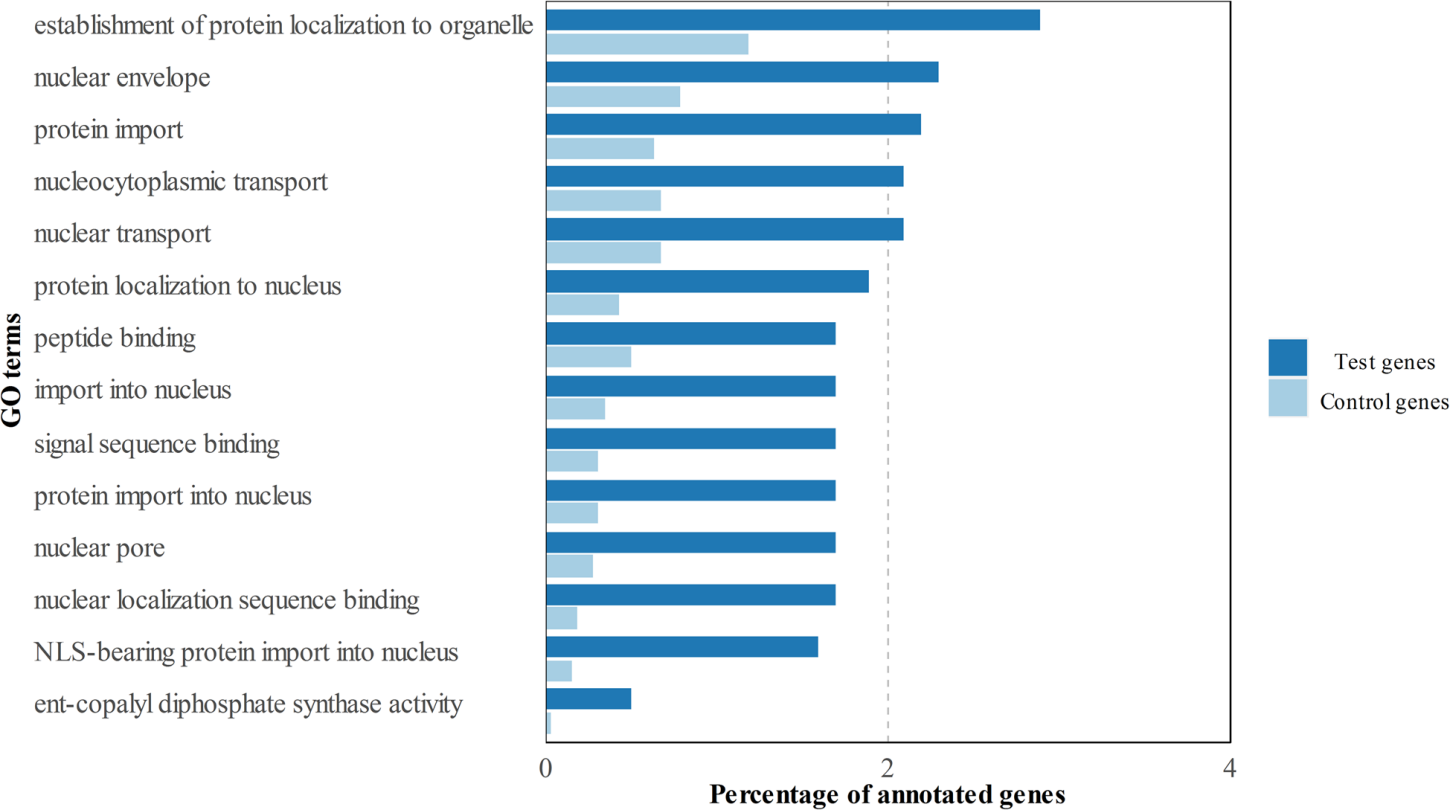
Enriched GO terms of the up-regulated genes in cultivars HR versus the rest of the groups

**Fig. 3:**
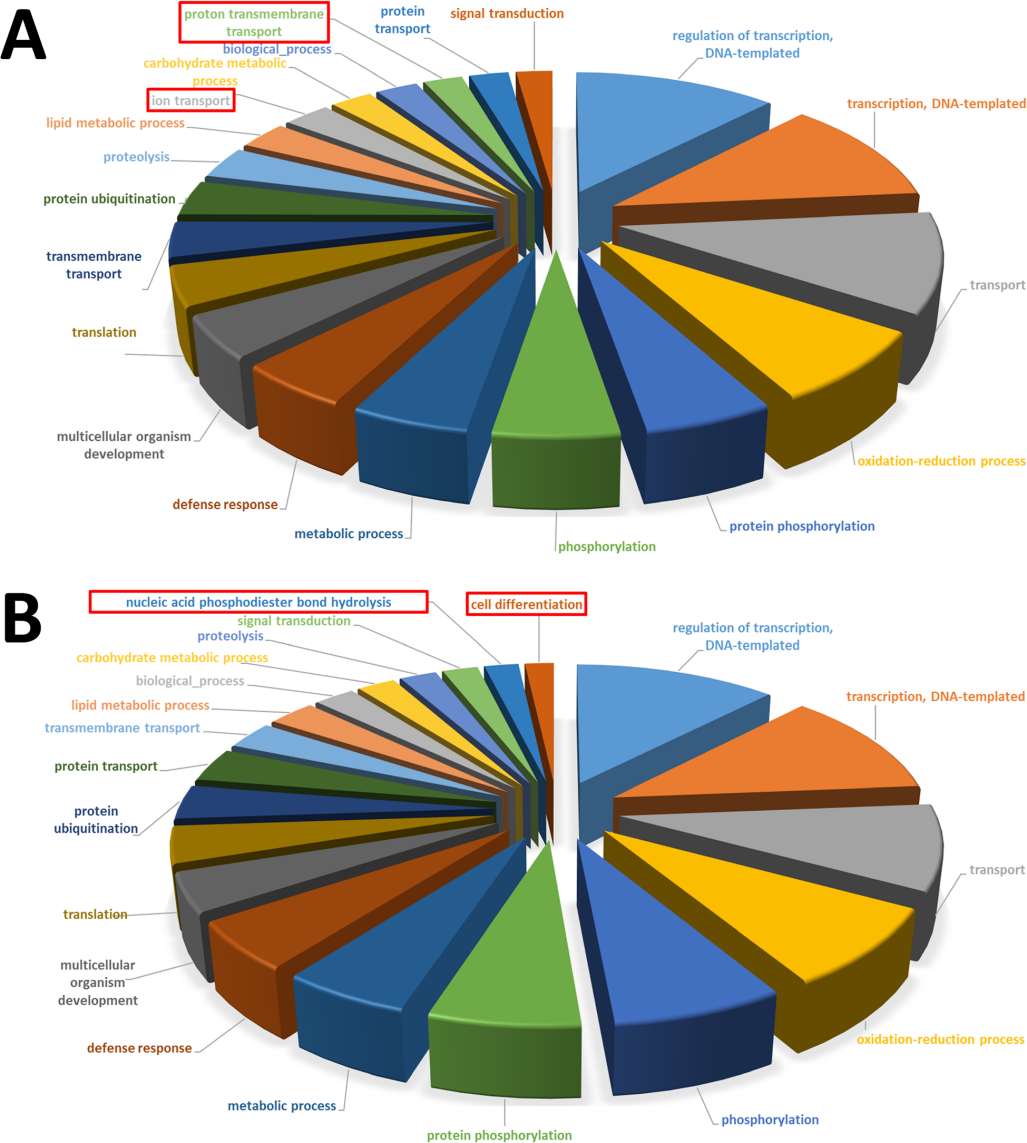
Top 20 Biological Processes at level 7 related with genes up regulated (**a**) and down regulated (**b**) in cultivars ES. Red boxes highlight terms that differ between both groups

**Fig. 4:**
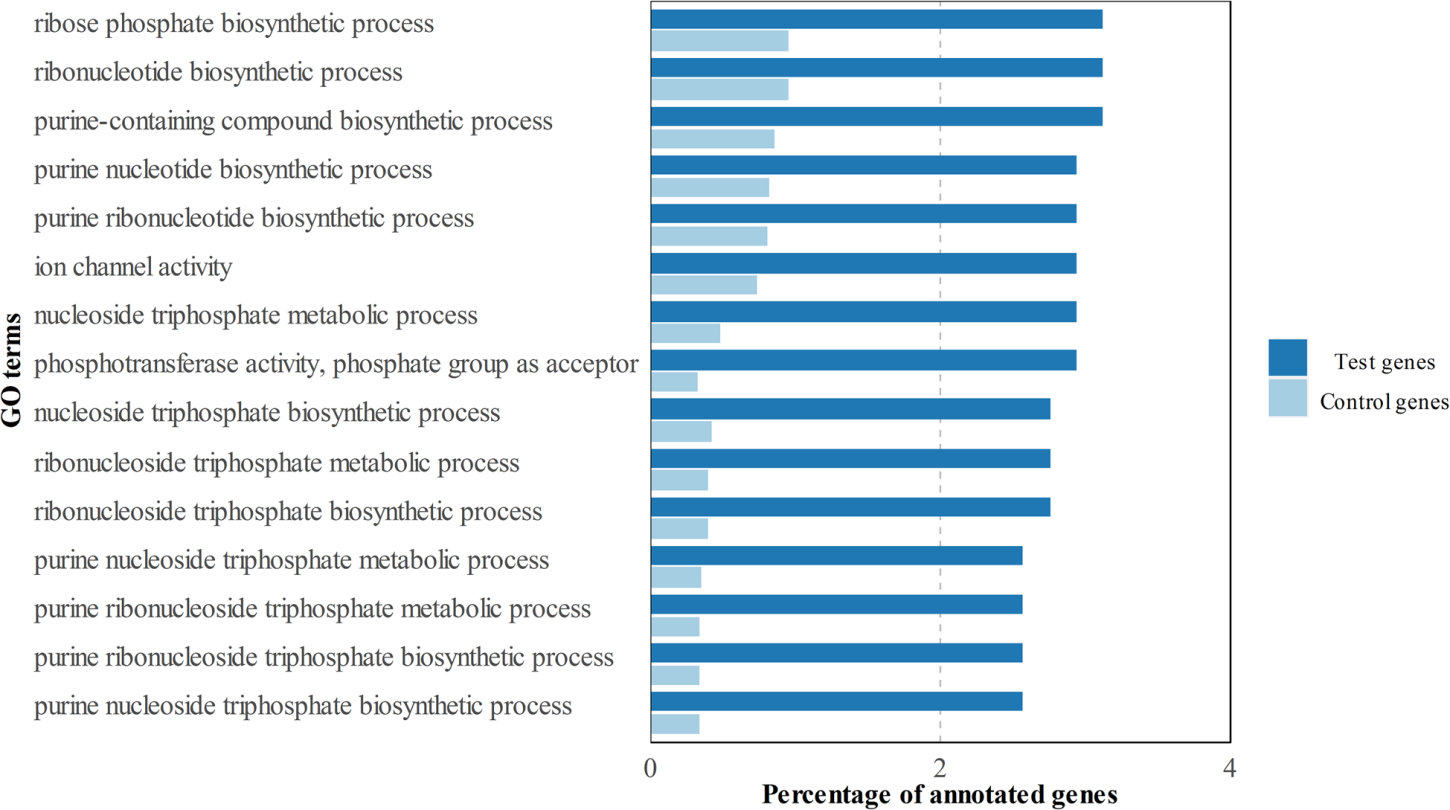
Top 15 Enriched GO terms of the up-regulated genes in cultivars ES versus the rest of the groups

**Fig. 5:**
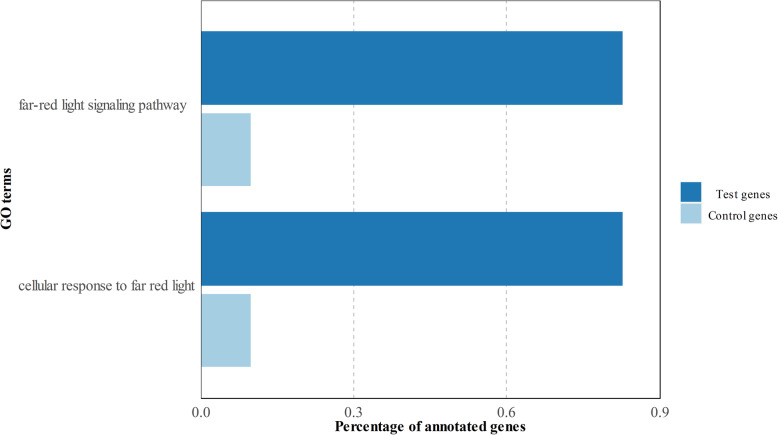
Enriched GO terms of the down-regulated genes in cultivars ES versus the rest of the groups

The comparison between the roots of cultivars ES with the other disease groups (HR-R-MS-S) resulted in 2606 differentially expressed unique genes. In this case, 914 genes were up-regulated and 1692 were down-regulated (see Additional file 2). Once again, the first approach with a GO direct count gave similar profiles in both gene sets (Fig. 3). However, the GO enrichment output of these root genes was quite informative. The ES up-regulated genes highlighted the strong relevance of biosynthetic processes in this tissue, with terms linked with purine processing, such as the purine-containing compound biosynthetic process (GO:0072522) or the purine ribonucleoside triphosphate biosynthetic process (GO:0009206), as well as an active energy metabolism, represented by several processes related to nucleosides triphosphate metabolism. The top 15 enriched GO terms are shown in Fig. 4. Despite the ES down-regulated genes almost doubling the up-regulated ones, only two terms were enriched in the first group. Both terms were related to far-red light (Fig. 5). Complete information about GO terms enrichment and the annotated genes can be consulted in Additional files (see Additional file 3).

### Opposite gene patterns between the HR and ES groups

**Fig. 6:**
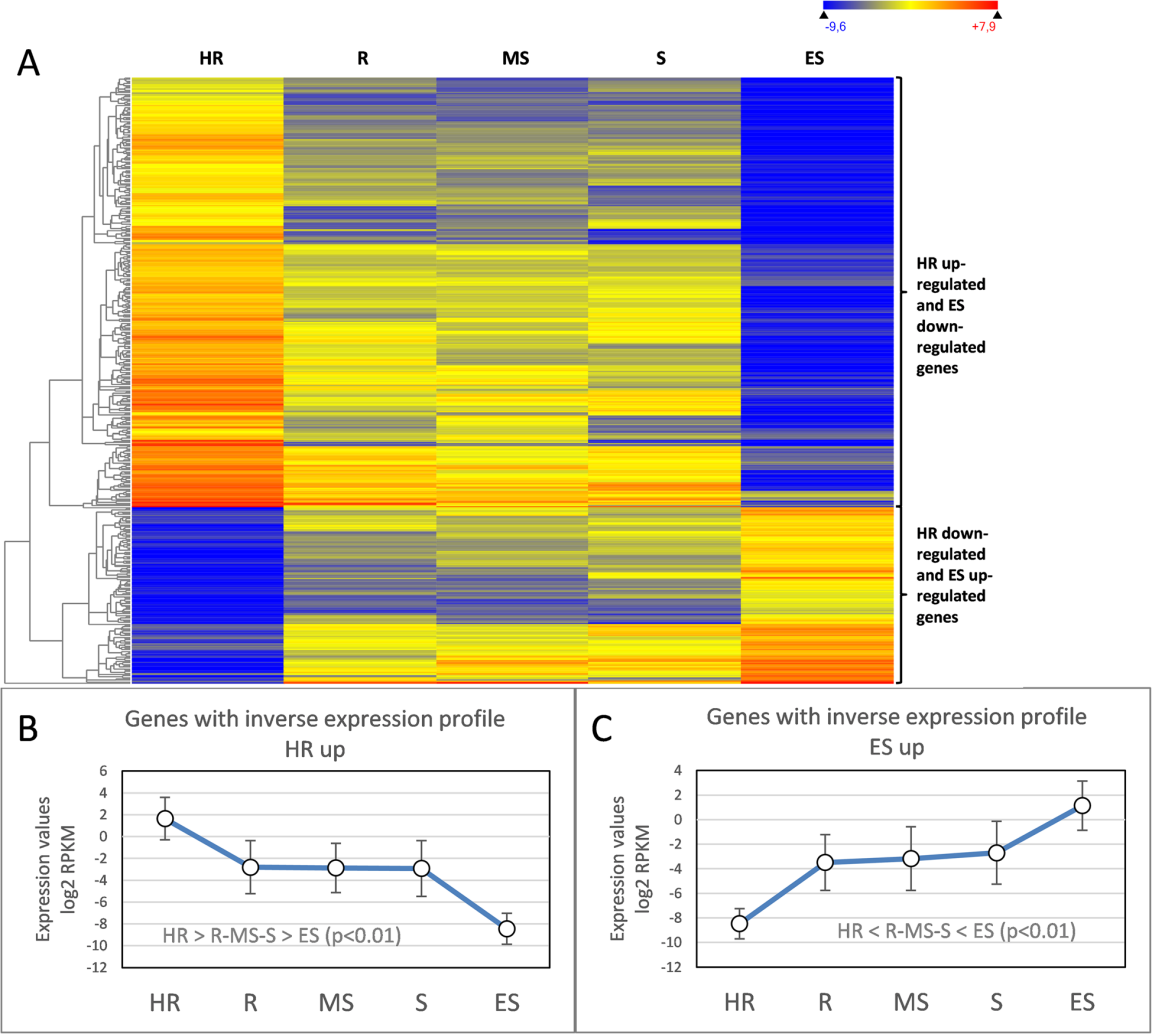
Genes that show an inverse expression profile from HR to ES cultivars. (**a**) Heat map. The gradient from red to blue represents the higher or lower gene expression value, respectively. (**b**) Trend line of genes overexpressed in HR cultivars and (**c**) overexpressed in ES cultivars. HR = Highly resistant; R = Resistant; MS = Moderately susceptible; S = Susceptible and ES = Extremely susceptible cultivars

When comparing the roots of cultivars HR and ES, 421 genes were found to be differentially expressed. Hence, the 299 genes up-regulated in the roots of group HR were down-regulated in the group ES, of which 218 were annotated (see Additional file 4). The opposite expression trend accounted for 122 down-regulated genes in the roots of group HR that were up-regulated in the ES group, with 83 unique genes correctly annotated (see Additional file 5). The overall set of 421 genes was selected by two different comparisons: HR versus the other groups and ES versus the other groups. This means that they present an expression pattern as HR > (R, MS, S) > ES, or the opposite one as HR < (R, MS, S) < ES (Fig. 6). This may eventually account for a significant role of this gene set in defining the resistance/susceptibility phenotype of the roots of cultivars HR and ES.

### Transcription factors

Transcription factors (TF) are key elements in the response to biotic and abiotic stresses. However, it is not well stablished how the TF are involved in the defense to *V. dahliae* infection. Twenty-nine TF coding genes were found to be up-regulated in the roots of cultivars HR and down-regulated in the ES ones. Of these, five CONSTANS-like genes containing the widely conserved CCT domain were found. They included three bZIP-like genes (two copies of *BZIP16* and one *BZIP44*), two auxin-responsive coding genes (*IAA14* and *IAA17*) and two ethylene (ET) response-related TF (*ERF070* and *DREB2C*) ones. The response of plants to this phytohormone could be crucial during *V. dahliae* infection as two TF from this family were found to also be affected, but with a higher expression in the ES roots (*ERF4* and *PLT2*). The TF group with higher expression levels in ES roots was smaller (13 unique genes), but quite interesting. Of these up-regulated genes, three genes coding for two-component response TF (*RR9*, *RR23* and *ARR8*) were found. A fourth component of this family was down-regulated in the roots of cultivars ES (*APRR2*). Therefore, this versatile TF group seems key in *V. dahliae* infection. As a huge amount of TF was up-regulated in HR and down-regulated in the roots of cultivars ES, and given the relevance of the nuclear transport highlighted by the GO analysis of the HR up-regulated genes, it would seem that the roots of cultivars HR possess highly active transcription activity. This process might be enhanced by the presence of seven importin coding genes, which were also up-regulated in the roots of cultivars HR and down-regulated in the ES ones.

### Defense-related genes

Twenty-four genes varyingly related to the immune response in plants showed higher expression values in the roots of HR, which were lower in ES. Up to four unique members were cytochrome P450 protein-coding genes (*CYP72A219*, *CYP76A2*, *CYP71D18, CYP71A8*), with a fifth component that was up-regulated in the roots of cultivars ES (*CYP704C1*). This is a family of monooxygenases involved in diterpene synthesis, a metabolite with anti-fungal activity. Another set of HR up-regulated and ES down-regulated genes was Germin-like protein-coding genes, classically linked with plant defense in response to biotic and abiotic stresses, but little is known about defense against fungi. Moreover, some independent unique genes down-regulated in the roots of cultivars ES proved crucial in plant immunity, such as Major pollen allergen Aln g 1, related to latex synthesis, or Polyamine aminopropyltransferase (*speE Saccharomyces* ortholog), involved in spermine synthesis through P450 cytochromes. Several immunity-related TF also had higher expression values in the HR group, but showed lower ones in the roots of cultivars ES. This was the case of the above-mentioned *bZIP* TF and nuclear transcription factor Y (*NFYA9*), also associated with defense response. This was also observed in several members of the pentatricopeptide repeat-containing protein family (*PCMP-E16*, *EMB2076*, *PCMP-H60*, *At2g01740*, *PCMP-H74*, At5g09450, *PCMP-H58*), which were up-regulated in HR, but down-regulated in ES. These are RNA-binding proteins that regulate gene expression at the RNA level.

### Development-related genes

A diverse group of genes related to root growth and development were found to be related to the resistance or susceptibility level. In fact, some of the genes that followed opposite expression patterns between the roots of HR and ES, and halfway in groups R, MS and S, participated in plant development. This group contains the five above-mentioned Cytochrome P450 genes, but also some more unrelated members, such as *LECRK42*. This gene was down-regulated in the roots of cultivars ES. It encodes a protein related to pollen development, which is also involved in plant immunity. Some are associated with root growth, such as *PHYB*, and presented higher expression levels in the roots of cultivars HR and control primary root growth through the far-red light response or *NSP2*, whose protein regulates striogalactone synthesis in this tissue. *SAG39* also appeared in this group, a protease-coding gene related to senescence and cell death that was up-regulated in the roots of cultivars ES and could also play a key role in the ET-auxins/gibberellin routes controlling root growth.

### Phytohormones-related genes

Another group of genes found to be differentially expressed had phytohormones response functions. The role of phytohormones was diverse in relation to the susceptibility to *V. dahliae* infection. In fact, some genes were up-regulated in the HR and other in the ES roots. For instance, some genes encoding the WAT1-related proteins family were grouped in this set, with three members down-regulated (2 copies of *At5g07050,* 1 of *At1g21890 Arabidopsis* orthologs) and two up-regulated (*At3g02690* and *At2g37450 Arabidopsis* orthologs) in ES roots. These proteins are vacuolar transporters of auxins and other phytohormones and are causally related to growth and elongation. Some other genes in this group code for splicing enzymes, such as helicase DEAH7 encoded by the gene *CUV,* which controls auxin-regulated development; a redox enzyme like PER47, which is a peroxidase that participates in auxin catabolism; a phospholipase, *PLC2*, linked with auxin biosynthesis. They were all down-regulated in the roots of cultivars.ES Besides, *SAUR36*, a gene that regulates auxin and gibberellins-mediated growth, showed higher expression levels in the roots of cultivars ES and lower levels in HR. Of the genes with an opposite pattern between HR and ES, and halfway in the other cultivars, seven unique entries coding for gibberellins-related proteins were found. Some are directly related to gibberellins, such as a soluble gibberellin receptor (*GID1B*), a gibberellin oxidase (*GA20OX1*) and a gibberellin-regulated protein (*GASA10*), and they all showed higher expression values in ES and lower ones in HR compared to the other cultivars. Furthermore, a catabolic dioxygenase of gibberellins was found to be less expressed in ES plants (*GA2OX1*), as was a gibberellins-related transcription factor (*EFM*). Two additional gibberellins-related genes showed down-regulation in HR roots and up-regulation in ES roots: a gibberellic acid transporter, the *NPF3.1* gene, and the aforementioned gene to encode senescence-associated protease (*SAG39*) that responds not only to ET, auxins and gibberellins, but also to abscisic acid (ABA). ABA signaling was also represented in this specific group of genes. In fact a receptor of this phytohormone (*PYL8*) showed lower expression levels in the roots of cultivars ES and a higher expression in HR roots, as well as C-terminal domain small phosphatase, a tetraticopeptide repeat protein, and two previously mentioned TF (*ERF070* and *DREB2C*). The only gene related to ABA to show up-regulation in ES roots was *HVA22*.

### Post-translational modification-related genes

Other genes with an opposite expression pattern between cultivars HR and ES were related to processes involved in protein post-translational modification. Some of them related to protein degradation and other to histone modification. In the first case, six genes implied in protein ubiquitination was highly represented. Hence five E3 ubiquitin-protein ligases were found, three up-regulated (*HRD1A*, *COP1* and *At4g11680 Arabidopsis* genes) and two down-regulated in the roots of cultivars HR (a *Hel2 Saccharomyces* ortholog and the *At3g02290 Arabidopsis* gene), but displayed the opposite trend in the ES ones. A single member of E2 ubiquitin-protein ligase (*UBC32*) was up-regulated in the roots of cultivars HR, but was down-regulated in the ES ones. Some genes participating in histone modification were also differentially expressed between the HR and ES comparisons to the remaining groups. Hence, three N-methyltransferases (the *ASHR3 Arabidopsis* gene, and the *Prmt3 Rattus* and *prmt6 Xenopus* orthologs) showed higher expression in the roots of cultivars HR and a lower expression in the ES ones. Another gene related to histones was also differentially expressed between the roots of cultivars HR and ES, the PHD finger protein-coding gene *AL5*, which showed lower expression levels in this last group.

## Discussion

The present work addresses how the gene expression profile in healthy roots might differ in cultivars depending on their susceptibility to *V. dahliae* infection, which would indicate that basic differences in the gene expression profile may account for disease resistance or susceptibility. An RNA-Seq of 29 adult olive cultivars was performed which were classified into the following categories: HR, R, MS, S and ES to Verticillium Wilt. Thus, quite a different gene expression pattern between the roots of cultivars HR and ES was observed compared to one another and the other disease response groups (R, MS, S). Although the age of the plant has been related to the defensive response to pathogens [14], the olive plants that we have used in this work can be considered as adult young plants of this centennial tree. So no differences in the gene expression profile can be attributed to differences in the age of the plants.

The first step to characterize the gene expression of each group was to conduct a GO terms enrichment analysis. It was found that most of the enriched terms involving the 1542 up-regulated genes in the roots of the HR cultivars were related in some way to the import of proteins to the nucleus. For instance, terms like nucleocytoplasmic transport, nuclear transport, import into the nucleus or nuclear localization signal (NLS)-bearing protein import into nucleus were observed. Indeed the NLS signal is the first and best-characterized molecular signal related to proteins whose final destination is the nucleus [15]. If we bear in mind that TF and other proteins linked with this process need to enter the nucleus to perform their biological functions, the massive protein mobilization to this organelle could explain, to some extent, the vast difference in gene expression terms between the roots of the HR cultivars and others. This ‘open access’ state of the nucleus in the root cells of cultivars HR to TF may also confer them an improved quicker response to stimuli like abiotic stresses or pathogen attack, which is what this work is about. In fact, several authors have widely discussed about the role of TF in plants defense response to *V. dahliae* [16, 17]. Intriguingly, only one out of the 14 GO terms enriched among the HR up-regulated genes was not related to protein transport: ent-copalyl diphosphate synthase activity. This enzymatic activity participates in gibberellins biosynthesis, phytohormones related to plant growth at different levels. Besides, two different transcripts coding for this enzyme have been observed in rice, of which one is involved in plant defense [18]. However, further studies are needed to depict the role of this enzymatic activity in Verticillium Wilt management by *O. europaea* plants.

Similarly, the up-regulated genes in the roots of cultivars ES clearly indicated energy metabolism, with a vast majority of terms related to active purine ribonucleoside triphosphate biosynthesis that might be linked to extremely active growth and development. As *V. dahliae* penetrates plants through root elongation sites [19], it is conceivable to think that active growing roots would be more prone to be infected by the pathogen, which would explain the most marked susceptibility displayed by these cultivars. This hypothesis is reinforced by the two GO terms that appeared among the down-regulated genes in the roots of cultivars ES: the far-red light signaling response and the cellular response to far-red light. In plants, this wavelength causes longer hypocotyls and shorter and less branched roots by reducing the effect of auxins signaling [20]. In fact, the *PHYB* coding gene is down-regulated in the roots of the ES cultivars*,* which is a photoreceptor that absorbs energy in the far-red light region to subordinate root growth to light and temperature [21]. Thus, according to these results, the roots of cultivars ES might not be sensitive to this specific signaling for growth inhibition, promoting further root growth compared to the remaining plant groups.

**Fig. 7:**
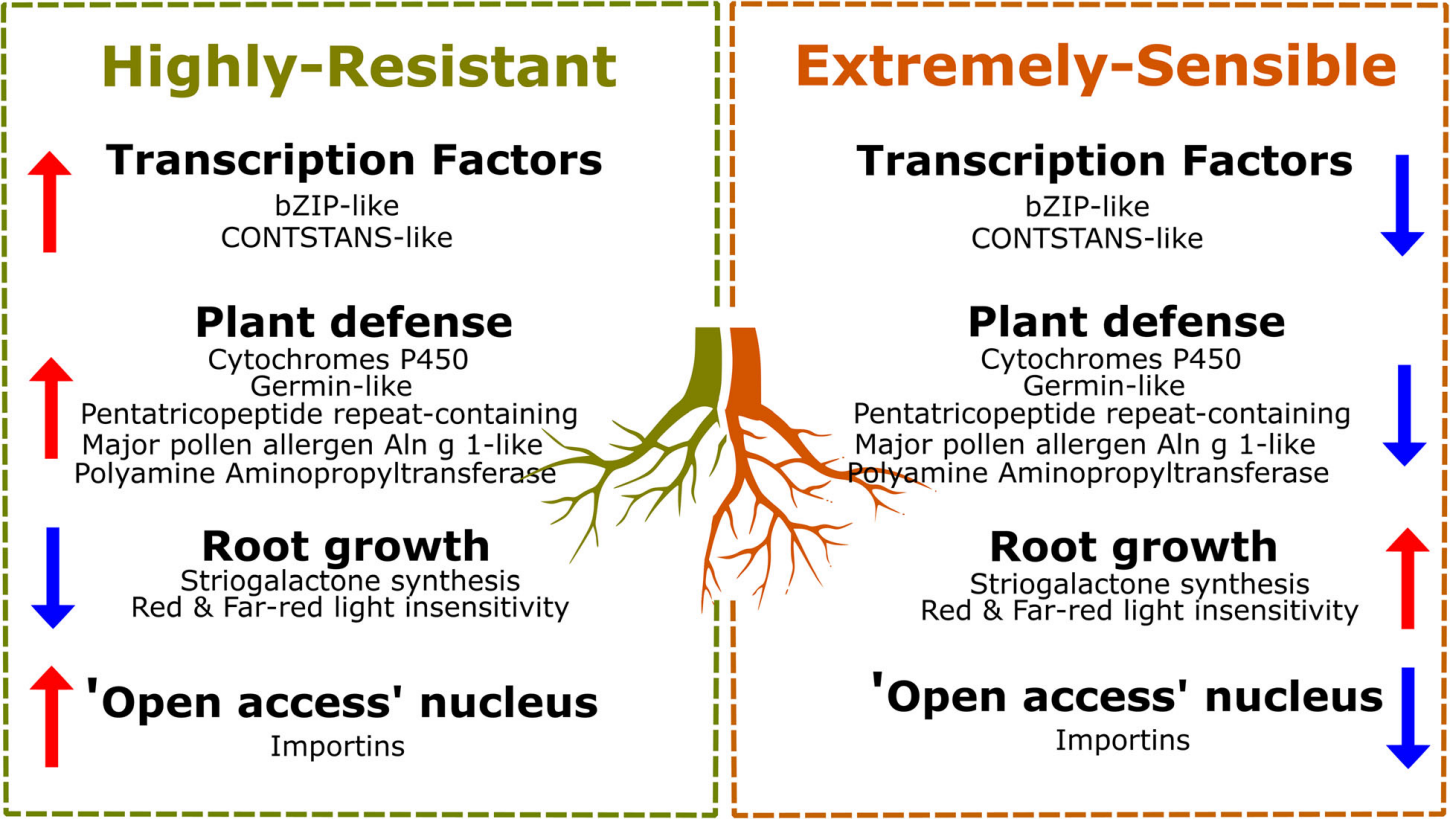
Proposed model summarizing the main gene expression findings in olive roots and the *V. dahliae* resistance phenotype

The higher expression in the roots of cultivars HR of *AL5* could reveal a clue: this gene encodes a PHD finger protein that recognizes and binds to the ‘Lys-4’ trimethylated tail of H3 histones, tagged as the start of transcription for virtually all transcriptionally active genes [22]. Therefore, several genes indicate that the roots of cultivars HR are the most active ones, particularly if we consider that at least 29 TF were up-regulated in them. Of these genes, the CONSTANS-like gene family was the most abundant, with five copies up-regulated in the roots of cultivars HR and down-regulated in the ES ones (Fig. 7). Although the role of these TF in plant resistance is unknown, CONSTANT genes were inhibited in soybean roots after nematode infection [23]. Other members of this family have been described as being essential in the far-red light response of plants growth by suppressing auxin response through the interaction with *PHYB* [24]. As previously mentioned, *PHYB* expression was also down-regulated in the roots of cultivars ES. Hence, the drastic fall in the expression of five gene copies of the CONSTANS-like genes could enhance the predicted insensibility of the roots of cultivars ES to far-red light-mediated growth inhibition (Fig. 7), which would provide a better understanding of the role played by auxins in the roots of cultivars ES -*V. dahliae* interaction. In line with this hypothesis, several TF involved in phytohormones response showed the same opposite root expression pattern between cultivars HR and ES, such as four ET response-related TF up- (*ERF070* and *DREB2C*) and down- (*ERF4* and *PLT2*) regulated in the roots of cultivars HR. Together with APETALA2 (*AP2*), ET-response factor (ERF) TF form the huge family of *AP2/ERF* genes composed by five groups depending on their number and the similarity of AP2/ERF domains [25]. Dehydration-responsive element (DRE)-binding proteins are one of these gene groups containing, among others, the *DREB2C* gene. These TF control water, high salinity and hot stress response and contain an ERF in their structure [25]. The *ERF070* transcription factor binds to the GCC-box pathogenesis-related promoter, which has been described to participate in the development of *Arabidopsis* roots [26]. The overexpression of an ERF transcription factor family member has been found to increase tomato resistance to *V. dahliae* infection, which acts as a promising ally to improve host resistance [27]. *ERF4,* one of the up-regulated genes in the roots of cultivars ES, is a transcriptional repressor that causes an ET-insensitive state and inhibits, among others, the expression of basic chitinase [28]. This enzyme hydrolyzes chitin, one of the major components of the fungal cell wall [29]. Besides *ERF4*, *PTL2*, an AP2-like transcription factor, was also up-regulated in the roots of cultivars ES. This gene is essential for establishing a stem cell niche in *Arabidopsis* roots [30] and ensuring adequate auxin signaling in root tips [31], the typical root growth-associated phytohormone. Hence, up to five copies of Walls Are Thin (WAT) 1-related protein coding genes were found: three members were up-regulated (2 copies of *At5g07050* and 1 of the *At1g21890 Arabidopsis* genes) and two were down-regulated (*At3g02690* and *At2g37450*) in the roots of cultivars HR, with the opposite expression trend in the ES ones. This protein family acts as closely related transmembrane transporters to the WAT proteins for vacuolar auxin transport [32] and cell-wall deposition [33]. These proteins also seem to confer broad-spectrum resistance to vascular pathogens throughout several mechanisms [34], such as increased lignin deposition observed in cotton roots in response to *V. dahliae* infection [35]. Although the WAT1-related protein appears to play some role in the olive cultivar roots-*V. dahliae* interaction, their erratic behavior in cultivars HR and ES suggests the need for more studies to fully understand their function. Among the genes somehow related to auxin signaling, the roots of cultivars ES showed lower expression levels of enzymes-coding genes, such as *CUV*, *PER47* or *PLC2*. The first is an RNA helicase that belongs to the DEAH family, a group of proteins involved in pre-mRNA splicing, but also performs additional functions like facilitation of auxin signaling-related genes [36] or the positive regulation of plant immunity against fungi [37]. The second one is a peroxidase that, as a redox-controlling enzyme, participates in a wide range of chemical processes like lignin biosynthesis, auxin metabolism, or even plant defense, depending on the expressed isoform and tissue. This specific isoform has been related to lignin synthesis in pears [38]. As previously reported in cotton [35], lignin accumulation has been described as an important mechanism against *V. dahliae* infection in olive roots [39]. Lastly, phospholipase *PLC2* is a positive regulator of auxin biosynthesis and has been recently described as a key gene in auxin-mediated root growth and development [40]. Although this result does not coincide with our findings about the role of auxins in the roots of cultivars ES, it could explain HR-ES differences by an interesting additional function of *PLC2*. Indeed, this protein is one of the earliest responses in plants when a microbe-associated molecular pattern is recognized by activating plant defense through reactive oxygen species production [41]. Thus, its down-regulation in the roots of cultivars ES might be linked to a low defense response in these plants instead of being related to auxin synthesis. Another auxin-related gene, *SAUR36*, was down-regulated in the roots of cultivars HR and up-regulated in the ES ones. This gene encodes Small Auxin Up RNA 36, which is related to hypocotyl elongation, whose expression is promoted by auxins and also responds to gibberellins [42]. The same expression pattern was found for telomerase activator *TAC1*, a gene that enhances auxin signaling [43]. This result makes more sense when coupled to the lower expression of two auxin-responsive coding genes (*IAA14* and *IAA17*) in the roots of ES vs. that in the roots of cultivars HR. It is well established that these TF act as repressors of auxin-mediated root growth in plants [44, 45]. As they are up-regulated in the roots of cultivars HR and down-regulated in the ES ones, auxin-responsive coding genes might be responsible for both impaired auxins signaling and a higher growth rate of the roots of the ES cultivars involved in *V. dahliae* infection.

Some genes also indicated the influence of gibberellin signaling on the different *V. dahliae* resistance degrees between the roots of cultivars HR and ES. Of them*, GA2OX1* encodes enzyme gibberellin 2-beta-dioxygenase 2, a deactivating enzyme of gibberellins that is expressed in roots [46]. Yet *GA2OX1* down-regulation may suggest a more active state of these phytohormones in the roots of cultivars ES. The pattern followed by the other gibberellins-related genes points in the same direction. For instance, a gibberellin-responsive cysteine protease (coded by *SAG39* gene) linked with leaf senescence in rice [47] showed a higher expression in the roots of cultivars ES and a lower expression in the HR ones compared to the remaining disease-resistant groups. This enzyme does not only respond to gibberellins, but also to ABA signaling [47]. In line with this, some of the above-mentioned TF related to ET (*ERF70* and *DREB2C*) showed opposite gene regulation in the HR and ES roots of cultivars (up- and down-regulation, respectively) and are also capable of responding to ABA. ERF TF possess dual functions by controlling the programmed cell death caused by ABA signaling and providing resistance to bacterial pathogens [48]. The main function of ABA is to manage plant response to biotic and abiotic stresses [49], but it also performs several secondary functions, such as growth regulation. For instance, lateral root growth inhibition by ABA recovers after *PYL8* gene expression [50]. Interestingly, the opposite expression pattern was observed between cultivars HR and ES, and was up-regulated in the roots of group HR. Similarly, C-terminal domains small phosphatase, involved in the negative regulation of ABA-mediated stress response in plants [51], was also up- and down-regulated in the roots of cultivars HR and ES, respectively.

Another group of relevant TF was the two-component response regulators family, which represents main actors in cytokinins signaling [52]. This TF controls plant development and growth through both cytokinins and ET responses [53]. *RR9* and *RR23* are components of this group which displayed an opposite expression pattern between the roots of cultivars HR and ES, which were up-regulated in ES. *RR9* was up-regulated in legumes when a symbiotic relation with soil bacteria was established, which suggests an important role for this gene in plant-microbiome interactions [54]. *ARR8*, also up-regulated in the roots of cultivars ES, is quickly induced by cytokinins, which is why it is considered a primary cytokinins response gene [55]. In fact, it has been demonstrated that a balanced signaling of these hormones could inhibit proliferation of *V. longisporum* in *Arabidopsis* [56]. A third member of two-component response regulators (*APRR2*) related to pigment accumulation in several fruits [57, 58] was down-regulated in the roots of cultivars ES, but up-regulated in the HR ones. The expression of this gene increases under drought conditions in potato plants and once again falls when plants are re-watered [59]. In these plants, the opposite expression pattern followed by the genes related to ERF, cytochrome P450, gibberellins, auxins and ABA highlights their role in response to these abiotic stresses [59]. These findings somehow mimic the results presented herein on the HR and ES cultivars in *V. dahliae* infection susceptibility terms*.* Although water stress can be proposed as one of the causes of this expression pattern, the fact that all the cultivars herein used were submitted to exactly the same conditions allows this hypothesis to be rejected. However, further studies are mandatory to assess the role of this set of genes in olive resistance to *V. dahliae*.

The response of olive against *V. dahliae* infection could be influenced by the interaction with other microorganisms, as previously observed in wild olives and between contrasting ‘Frantoio’ and ‘Picual’ cultivars [60, 61]. This olive-associated microbiome could, in turn, be affected by phytohormones like striogalactones [62], whose synthesis could be regulated by the *NSP2* gene [63] down- and up-regulated in the roots of cultivars HR and ES, respectively (Fig. 7). Thus striogalactone could sway the disease response by enhancing root growth [64] or managing the relations between plant roots and soil microbiome. Different gene expression patterns were observed in the genes involved in the development of several plant organs. Hence, *LECRK42* is necessary for pollen development, but also plays a key role in immunity as knock-out models of *Arabidopsis* for this gene are more susceptible to *Phytophthora* infection [65]. However, the most numerous group of genes related to development and immunity was the cytochrome P450 protein family, a versatile group of monooxygenases with four members up-regulated in the roots of cultivars HR and down-regulated in ES ones, and one (*CYP704C1*) that follows the opposite pattern. Therefore, depending on the family member, both the overexpression and silencing of these genes could enhance the resistant response against *V. dahliae*. Indeed *CYP77A2* overexpression increased resistance against *V. dahliae* thanks to its role in the biosynthesis of antifungal compounds [66], whereas the down-regulation of *CYP94C1*, which is highly expressed in plant roots, improves resistance to Verticillium wilt in cotton [67]. Of the HR up-regulated cytochromes P450, two members of CYP71 and one of CYP72 were found, which are precisely two of the three families of the cytochromes P450 monoxygenases involved in diterpene biosynthesis [68]. The third HR up-regulated member was a CYP76 cytochrome that specializes in labdane diterpene metabolism [68]. These are one of the most important plant metabolites to participate in development and defense. In plant roots, diterpenes are up-regulated by fungal endophytes to confer host resistance [69]. Given their wide variety of roles, unveiling how they act exactly is no easy task. It seems logical to suspect that cytochromes P450 have some relevance in conferring *V. dahliae* resistance to the roots of cultivars HR (Fig. 7). Further research is needed to understand if they influence only plant development, plant immunity, or both.

Of those genes with opposite expression patterns between the roots of cultivars HR and ES, a few of them are closely related to plant defense mechanism. Hence a huge amount of pentatricopeptide-repeat containing protein-coding genes displayed contrasting expression pattern in the roots of cultivars HR and ES; i.e. up- and down- regulated, respectively (Fig. 7), including five widely expressed isoforms (*PCMP-E16*, *EMB2076*, *PCMP-H60*, *At2g01740*, *PCMP-H74*) and two organelle-specific ones: one from mitochondria (*At5g09450*) and one from the chloroplast (*PCMP-H58*). These are RNA-binding proteins expressed mainly in organelles, but also in the nucleus, that regulate gene expression at the RNA level thanks to their ability to splice, stabilize, edit and translate this molecule [70]. With such a wide range of potential action points, it would be difficult to accurately decrypt how pentatricopeptide-repeat containing proteins act in *V. dahliae* resistance. Nonetheless, it has been described how some of these genes generate phased small interferences RNA (phasiRNA) as a defense response to fungal infection in soybean plants [71]. Furthermore, three genes from subfamily 1 from germin-like protein, unlike ES roots, were up-regulated in HR ones. As these genes have been characterized as protective against bacterial and fungal infections in several plants [72, 73], their lower expression could be an additional weakness in the roots of cultivars ES. This circumstance could be even worse combined with the higher expression levels of the three found bZIP TF, i.e. two copies of *BZIP16* and one of *BZIP44* genes, in the roots of cultivars HR, and lower ones in cultivars ES compared to the other disease response groups (Fig. 7). This result is relevant if we take into account that they belong to one of the six major families of TF linked with abiotic and biotic responses [74]. By way of example, some members of this family activate defense genes in *Arabidopsis* in response to fungal attack by conferring basal defense and disease resistance [75]. A polyamine aminopropyltransferase gene was also down-regulated in the roots of cultivars ES (*spE Saccharomyces* ortholog; Fig. 7). In plants, these enzymes participate in spermidine synthesis, an intermediate metabolite for the spermine biosynthetic pathway. Accordingly, it has been described that spermine is capable of triggering the so-called hypersensitive response-mediated resistance to pathogens by plants [76]. Furthermore, the protective role of spermine is mediated by the oxidative response, managed mainly by polyamine oxidase enzymes [77]. It is noteworthy that the *Arabidopsis* mutants overexpressing these enzymes have improved resistance to *V. dahliae* infection due, for other reasons, to the participation of MAP kinases and cytochromes P450 [78]. The last family of genes was also down-regulated in the roots of cultivars ES. As spermine is a strong activator of the plant defense response, it could be hypothesized that this ‘ready-to-go’ state in spermine biosynthesis could provide the roots of cultivars HR with extra defense.

## Conclusions

Altogether, our results suggest that the expression pattern followed by a vast number of genes (and the roles they can play) could point out a different scenario in the roots of ES cultivars compared to HR ones. Hence, it seems that the gene expression in the roots of uninfected ES cultivars focuses more on growth and development, while some other functions like defense against pathogens have a higher expression level in uninfected HR cultivars, which would thus influence the level of resistance to a potential *V. dahliae* infection.

## Methods

### Plant material

Root samples of 58 adult plants corresponding to 29 olive cultivars (two biological replicates each) were collected at the World Olive Germplasm Bank at IFAPA Centro ‘Alameda del Obispo’ (37.8587539,-4.8012045, Córdoba, Spain) in spring 2017 (See Additional file 6) [79]. The olive trees under study were all adults, in productive stage and in good phytosanitary conditions without any disease symptoms. Their age ranged from 14 to 20 years old. They were randomly planted at 7 × 7 m spacing in alkaline loam and sandy-loam soil. Samples were obtained around 30 to 40 cm from the trunk and were immediately washed and immersed in liquid nitrogen. Only tip fragments no larger than 10 cm were taken. Frozen root tissues were disrupted in two pulverization rounds with a Retsch® Mixer Mill 400 using 20 mm and 5 mm steel beads, respectively. This step provided the frozen fine powder needed for RNA extraction.

### RNA-Seq in olive tree roots

To extract RNA, 0.1 g of frozen powdered roots was immediately processed by the Spectrum^TM^ Plant Total RNA Kit (Sigma-Aldrich, St. Louis, MO, USA) by treating all the samples by on-column DNAse I digestion (Roche, Basel, Switzerland). Prior to RNA sequencing, the quality and quantity of samples were determined by a Bioanalyzer 2100 with an RNA 6000 nano assay (Agilent Technologies, Santa Clara, CA, USA). Afterward, poly(A) + RNA was isolated on poly-T oligo-attached magnetic beads to obtain suitable mRNA templates to perform reverse transcription. cDNA was PCR-amplified to obtain double stranded cDNA libraries. The quality of these libraries was checked in a TapeStation 4200 with a high sensitivity bioassay (Agilent Technologies). Finally, cDNA libraries were sequenced by paired-end sequencing (100 × 2) in an Illumina HiSeq2500 sequencer in the rapid mode and running two different lines in the flow cell as technical replicates for each sample. RNA-Seq processing was performed by Sistemas Genómicos (Valencia, Spain).

### Raw data processing

Raw reads fastq files were preprocessed in two steps. First, FastqMcf from ea-utils [80] was used to discard adaptors, as well as poor quality or short reads and unknown nucleotides. The minimum quality threshold was set at 30 and the minimum length at 50 bp (Q30L50). Second, another quality control of sequencing process was conducted by NGS QC [81], an even more detailed statistics were obtained to properly adjust the trimming and cleaning parameters for the Trim Galore software (https://github.com/FelixKrueger/TrimGalore).

### Gene expression and ontology analysis

The expression analysis was performed by the DNAStar (ArrayStar 15) Qseq software for RNA-Seq analyses (www.dnastar.com), with Oleur061 taken as the reference genome [82]. To map reads, the k-mer value was established at 63 and a minimum of 95% of matches was required. Values were expressed as Reads per Kilobase Million (RPKM) and 0.1 RPKM was the threshold value to consider a gene to be expressed. Furthermore, a fold change (FC) of 8 or higher with a Benjamini-Hochberg adjusted *p* value of 0.01 (*False Discovery Rate*, FDR < 1%) was applied to assume differential expressions among groups of disease responses. These groups distinguished the Extremely Susceptible (ES), Susceptible (S), Moderately Susceptible (MS), Resistant (R) and Highly Resistant (HR) cultivars (see additional file 6) [79]. To assess the biological relevance of the differentially expressed genes, a Gene Ontology (GO) analysis was conducted in two steps with the OmicsBox software, v.1.2 [83]. First, a direct GO count at level 7 was applied to sum the biological processes (BP) linked with each gene set. Second, a GO enrichment analysis based on a two-tailed Fischer’s Exact Test with an FDR lower than 5% was conducted to assess the enriched terms compared to the control group. For this purpose, the genes showing an expression (≥ 0.1 RPKM) in any root sample were defined as the control group to be compared to.

## Supplementary Information

**Additional file 1.** Expression values of differentially expressed genes in cultivars HR.

**Additional file 2.** Expression values of differentially expressed genes in cultivars ES.

**Additional file 3.** Full list of statistically significant GO terms of cultivars HR and ES.

**Additional file 4.** HR up-regulated genes among those with an opposite pattern expression between HR and ES roots (HR > (R, MS, S) > ES).

**Additional file 5.** HR down-regulated genes among those with an opposite pattern expression between HR and ES roots (HR > (R, MS, S) > ES).

**Additional file 6.** Table of groups of olive cultivars according to Verticillium wilt resistant response.

## Data Availability

The datasets generated during the current study are available in the Gene Expression Omnibus (GEO) repository, accession number GSE152236 (https://www.ncbi.nlm.nih.gov/geo/query/acc.cgi?acc=GSE152236). The plant material is stored at Department of Experimental Biology, Center for Advanced Studies in Olive Grove and Olive Oils, University of Jaén, Jaén, 23071, Spain.
