# Supplementary material for: Verticillium wilt resistant and susceptible olive cultivars express a very different basal set of genes in roots
Source: BMC Genomics. 2021 Apr 1;22:229. doi: 10.1186/s12864-021-07545-x (PMC8017696; doi:10.1186/s12864-021-07545-x)
Supplement: Supplementary file 6 — Additional file 6. Table of groups of olive cultivars according to Verticillium wilt resistant response. [file 12864_2021_7545_MOESM6_ESM.docx]

Additional file 6 | Table of groups of olive cultivars according to verticillium wilt resistant response.

| **Highly Resistant (HR)** | |
| --- | --- |
|  | R80 - Frantoio |
|  | R757 - Manzanillera de Huercal Overa |
| **Resistant (R)** | |
|  | R539 - Dokkar |
|  | R218 - Koroneiki |
|  | R82 - Leccino |
|  | R1125 - Maarri |
|  | R699 - Maureya |
|  | R95 - Uslu |
| **Moderately Susceptible (MS)** | |
|  | R231 - Arbequina |
|  | R711 - Barnea |
|  | R1026 - Barri |
|  | R1141 - Fishomi |
|  | R1812 - Klon 14 |
|  | R21 - Manzanilla de Sevilla |
|  | R108 - Lianolia Kerkyras |
|  | R224 - Morrut |
|  | R700 - Myrtolia |
|  | R9 - Picual |
|  | R3 - Picudo |
|  | R969 - Piñonera |
|  | R51 - Verdial de Vélez Málaga |

| **Susceptible (S)** | |
| --- | --- |
|  | R842 - Abbadi Abou Gabra |
|  | R1043 - Abou Salt Mohazam |
|  | R118 - Chemlal de Kabylie |
|  | R226 - Llumeta |
|  | R669 - Menya |
|  | R358 – Temprano |
| **Extremely Susceptible (ES)** | |
|  | R1118 - Jabali |
|  | R345 - Mastoidis |
| *Olive cultivars classification according to *V. dahliae* resistance evaluation [74]. Names and register numbers of cultivars from The World Olive Germplasm Collection of IFAPA (Córdoba). | |
